# Comprehensive Analysis of lncRNAs, miRNAs and mRNAs in Mouse Hippocampus With Hepatic Encephalopathy

**DOI:** 10.3389/fgene.2022.868716

**Published:** 2022-05-05

**Authors:** Huijie Zhang, Wenjun Zhang, Guangyin Yu, Fang Li, Yuqing Hui, Shuhan Cha, Meiying Chen, Wei Zhu, Jifeng Zhang, Guoqing Guo, Xiaobing Gong

**Affiliations:** ^1^ Department of Anatomy, Neuroscience Laboratory for Cognitive and Developmental Disorders, Medical College of Jinan University, Guangzhou, China; ^2^ Department of Gastroenterology, The First Affiliated Hospital of Jinan University, Guangzhou, China; ^3^ Nursing School, Jinan University, Guangzhou, China

**Keywords:** hepatic encephalopathy, long non-coding RNA, microRNA, Six3os1, AQP1, cognitive function

## Abstract

Hepatic encephalopathy (HE) often presents with varying degrees of cognitive impairment. However, the molecular mechanism of its cognitive impairment has not been fully elucidated. Whole transcriptome analysis of hippocampus between normal and HE mice was performed by using RNA sequencing. 229 lncRNAs, 49 miRNAs and 363 mRNAs were differentially expressed in HE mice. The lncRNA-miRNA-mRNA interaction networks were established, Gene Ontology (GO) and Kyoto Encyclopedia of Genes and Genomes (KEGG) pathway analyses were performed. Dysregulated RNAs in interaction networks were mainly involved in synaptic plasticity and the regulation of learning and memory. In NH4Cl-treated hippocampal neurons, the dendritic spine density and maturity decreased significantly, the amplitude and frequency of mIPSC increased, while the amplitude and frequency of mEPSC decreased. These manifestations can be reversed by silencing SIX3OS1. Further research on these no-coding RNAs may lead to new therapies for the treatment and management of brain dysfunction caused by HE.

## 1 Introduction

Hepatic encephalopathy (HE) is a complex neurological syndrome brought on by hepatic dysfunction. Patients present with varying degrees of attention deficit, cognitive impairment, and psychomotor abnormalities. Moreover, the morbidity of HE increases annually, seriously affecting the quality of life and prognosis of patients (Wijdicks, 2017). Hyperammonemia is one of the significant drivers of cognitive impairment in HE. Accumulation of glutamine in astrocytes as a result of hyperammonemia alters glutamate-glutamine metabolism leading to excitotoxicity and subsequent neuronal dysfunction (El-Marasy et al., 2019). Hyperammonemia impairs synaptic plasticity as well as novelty acquisition in corticostriatal and hippocampal pathways. These pathways are involved in learning behavior and goal-directed (Chepkova et al., 2017; Stravitz et al., 2018). Patients with HE also presents with neuropsychiatric symptoms such as anxiety and fatigue, in which basal ganglia dysfunction plays a role (Mendez et al., 2008). The related molecular mechanism was partially clarified at the protein level, but the upstream molecular mechanism is still unclear, especially the roles of non-coding RNAs(ncRNAs).

ncRNAs play significant gene-regulatory roles, coordinating many biological functions, which play crucial roles in the nervous system disease (Ma et al., 2020). Overexpression of MALAT1 can increase SIRT1 expression by inhibiting miR-142-3p, thus improving cerebral ischemia-reperfusion injury and cognitive dysfunction (Meng et al., 2021). LncRNA 4344 silencing can down-regulate the expression of NLRP3 by targeting miR-138-5p, attenuated LPS-induced neuroinflammation, and alleviate cognitive dysfunction (Feng et al., 2021). Previous studies have shown that lncRNA differentially expressed in serum of patients with Mild Hepatic Encephalopathy, and those differentially lncRNA play important roles in regulating inflammatory and immunological profiles (Wang et al., 2021).

In order to systematically understand the regulation mechanism of HE-induced cognitive dysfunction, we analyzed the expression profiles of lncRNAs, miRNAs, and mRNAs in the hippocampus tissues of normal and HE mice by RNA sequencing (RNA-seq). We analyzed the function of differentially expressed RNAs, constructed IncRNA-miRNA-mRNA interaction networks. In the networks, AQP1 is one of the most significant genes, and we also noticed that previous research showed that AQP1 silencing attenuates the cognitive impairment in Alzheimer disease (AD) through activation of the Wnt signaling pathway (Yu et al., 2020). The upstream gene of AQP1 was predicted to be SIX3OS1. Therefore, we also explored the role of lncRNA SIX3OS1. The results suggested that SIX3OS1 might regulate the expression of AQP1 by targeting miR-743b-3p. These are involved in dendritic spines development and synaptic function, leading to impaired learning and memory. Our results will be crucial in guiding the future research of the molecular mechanisms of epigenetics underlying HE cognitive dysfunction from the perspective of lncRNAs or miRNAs and help identify new therapeutic targets.

## 2 Material and Methods

### 2.1 Preparation and Identification of HE Mouse Model

The healthy male C57BL/6J mice (6 weeks) were purchased from Guangdong Experimental Animal Center (Guangzhou, China). Keep food and water free in an environment with regular light time (12 h/day) and relative humidity of 50%–70%. The mice acclimated to the environment for 7 days. Then, seventeen mice were randomized into two groups, nine mice were injected intraperitoneally with thioacetamide (HE group) and eight mice from the control group (Ctrl group). Dilute the thioacetamide powder (TAA; Sigma Aldrich, United States) to 10 mg/ml with normal saline when it will be used. The drug dosage was determined according to the body weight of the mice at the time of administration. The HE group received 100 mg/kg thioacetamide intraperitoneal injections daily on the first and second days, and then 50 mg/kg TAA daily from the third to the seventh day (Sun et al., 2019). Control group were treated with an equal volume of normal saline. Following the completion of the model building, the successful construction of HE mice were confirmed by liver pathological examination and behavioral examination. Hippocampus tissues from HE mice and ctrl mice (*n* = 3) were used for the analysis of RNAs, miRNAs and mRNAs. The animal experiments were approved by the ethics committee of Jinan University.

#### 2.1.1 Behavioral Test

For each test, mice acclimated 30 min in the testing room. A light intensity of 150 lux was presented during adaptation and throughout the testing periods. All assays were performed at the same time of day and there must be control and HE groups for each test.

##### 2.1.1.1 Open Field Test

Locomotor activity was measured as described previously (Wang et al., 2018). In brief, the mouse was placed in a chamber (50 cm × 50 cm × 10 cm). Movement in the chamber was monitored for 10 min using an overhead camera and tracking software (EthoVision; Noldus).

##### 2.1.1.2 Morris Water Maze Test

In order to evaluate the spatial learning function, the MWM test was performed as previously described. (Velazquez et al., 2019; Velazquez et al., 2020). The water maze test room was always kept at 23–25°C, and each mouse was tested in a circular water tank with a diameter of 1.2 m. The platform (9 cm in diameter) was submerged 1 cm below the water surface. Milk was added to opaque the water and hide the platform. Each mouse was tested 4 times daily during five consecutive days. The day before the first day of training, the animals were subjected to two 1-min trials without a platform to allow them to adapt to the maze. For all mice, the platform position was retained in the same quadrant. On the first day of training, the animals were randomly released from one of the three fixed points on the edge of the water tank and allowed to swim freely for 1 min, or until a platform was found and escaped the swimming task. If they could not find the platform, the experimenter would guide them to the platform. Let them stay on the platform for 15 s. On the sixth day, the mice underwent another test called the “spatial probe test”. In this task, the platform was removed, and each mouse was allowed to swim freely within 1 min. All trials were video recorded, and finally, the escape latency of each mouse and the time spent in the target quadrant were recorded.

##### 2.1.1.3 Elevated Plus Maze Test

The elevated maze test was used to detect anxiety in mice. The installation is 50 cm above the ground and consists of four perpendicular arms, two open arms (30 cm × 5 cm) and two closed arms (30 cm × 5 cm). At the beginning of the experiment, the mouse was gently placed in the central area of the maze facing the open arm and allowed to explore freely for 10 min. The video tracking software Topscanlite 3.0 was used to record and analyze the residence time of the mice in the open and closed arms, the number of times they entered the open arms and the total distance of movement. The shorter the time the mice stayed in the open arm, the fewer times the mice entered the open arm, which represented more severe anxiety.

##### 2.1.1.4 Y Maze Test

The Y-maze consists of three arms at an angle of 120° and can be used to evaluate mice’s spatial working memory ability. At the beginning of the experiment, mice were gently placed at the end of one arm and allowed to explore freely for 5 min. The video tracking software Topscanlite 3.0 was used to record the sequence and total number of times the mice entered each arm. When the mice entered different arms for three consecutive times, it was recorded as a correct alternating response, and then the alternating rate was counted.

##### 2.1.1.5 Novel Object Recognition Test

The novel object cognition experiment was used to assess the short-term memory ability of mice based on the mice’s preference for novel objects. First, the mice were placed in a 40 cm × 60 cm × 40 cm test box without a lid and allowed to move freely for 5 min; then two objects of the same color, size and material were placed in symmetrical positions in the test box, the mice were placed in the test box and allowed to explore freely for 5 min. After an interval of 1 h, the test phase was carried out. One of the objects was replaced with another new object of different color and shape, and the position was kept unchanged, and the mice were again put into the test box to explore for 5 min. The video tracking software Topscanlite 3.0 was used to record and analyze the sniffing time and times of old and new objects in the second stage.

##### 2.1.1.6 Three-Chamber Social Interaction Test

A three-box social experiment was used to assess the social ability of mice. The experiment was carried out in a three-chamber transparent rectangular box, with a channel in the middle to connect the three chambers, and a mesh cage that could accommodate one mouse was placed in each of the two chambers. Adaptation stage: The test mice were placed in the middle of the box and allowed to explore freely for 10 min; Social preference stage: A stranger mouse (Stranger 1) of the same species and sex were placed in a mesh cage on one side, and the test mice were allowed to explore freely for 10 min; social novelty stage: a second stranger mouse of the same sex (Stranger 2) was placed in the other side of the mesh cage, and the test mice were allowed to explore freely in the three-chamber for 10 min. The video tracking software Topscanlite 3.0 was used to record and analyze the contact time, social preference index and social novelty index of the tested mice with Stranger 1 and Stranger 2.

#### 2.1.2 Hematoxylin-Eosin Staining

On day 16, the behavioral test was completed, then the mice were sacrificed at day 17. The mice’s liver tissues were fixed in 4% paraformaldehyde (Meilunbio, MA0192). After embedding in paraffin, making paraffin sections (5-6 um) and then staining with hematoxylin and eosin according to the standard protocol (Guo et al., 2017). Pathological changes of the liver tissues were observed and photographed under the light microscope.

### 2.2 RNA Extraction, Sequencing and Identification of Differentially Expressed RNA

#### 2.2.1 LncRNA and mRNA Sequencing and Identification of Differentially Expressed LncRNA and mRNA

According to the manufacturer’s protocol, the total RNA was extracted from the mouse hippocampus using a commercial RNA isolation kit (Thermo Fisher Scientific, United States). RNA quality was assessed on a Bioanalyzer (Agilent Technologies, United States) and checked using a 1% agarose gel electrophoresis. After the total RNA was extracted, the ribosomal RNAs (rRNAs) were removed to retain mRNAs and ncRNAs. The enriched mRNAs and ncRNAs were fragmented into short fragments and turned into cDNA *via* reverse transcription and random primers by using fragmentation buffer. Subsequently, the second-strand cDNA was synthesized by mixing DNA polymerase I, RNase H, dNTPs and buffer. Immediately thereafter, cDNA-fragments were purified using PCR extraction kit (Qiagen, Netherlands), end-repaired, poly (A) tails added, and ligated with Illumina sequencing adapters. The second-strand cDNA was digested using Uracil-N-Glycosylase. All digested products were visualized by 1% agarose gel electrophoresis and amplified by PCR, and sequenced using an Illumina HiSeqTM 4000 by Gene Denovo Biotechnology Co. (Guangzhou, China).

To get high quality clean reads, reads were further filtered by fastp (version 0.18.0). All subsequent analyses are performed using clean reads. Transcripts were assembled with the Stringtie software (http://ccb.jhu.edu/software/stringtie/, version 1.3.4) which together with HISAT2 software (version 2.1.0), allow biologists to identify new genes and new splice variants of known ones. Transcripts abundances were quantified by software Stringtie in a reference-based approach. For each transcription region, a FPKM (fragment per kilobase of transcript per million mapped reads) value was calculated to quantify its expression abundance and variations, using RSEM software. Then, two softwares CNCI (version 2) and CPC (version 0.9-r2) (http://cpc.cbi.pku.edu.cn/) were used to predict the protein-coding potential for new parameters. The intersection of both non protein-coding potential results was chosen as long non-coding RNAs. Finally, the differentially expressed transcripts of coding RNAs and ncRNAs were analyzed, respectively. RNAs and ncRNAs differential expression analysis was performed by DESeq2 software between two different groups (and by edgeR between two samples). lncRNAs and mRNAs with a fold change ≥2 and a false discovery rate (FDR) < 0.05 in a comparison as significant differentially expressed genes. Differentially expressed mRNAs were then subjected to enrichment analysis of GO functions and KEGG pathways.

#### 2.2.2 miRNA Sequencing and Identification of Differentially Expressed miRNA

Total RNA was extracted from the control and HE samples using a RNA extraction kit (Invitrogen, United States). The 18–30 nt RNA fragment was recovered by PAGE gel electrophoresis, the 3′ adaptors and the 5′ adaptors were connected to the small RNA with T4 ligase successively, and then RT-PCR and PCR were performed on the small RNA with the two-sided linkers. Finally, the band of about 140–160 bp was recovered and purified by PAGE electrophoresis. Two sets of RNA samples with high integrity and purity were selected to construct a small RNA library. Then, using Agilent 2100 and qPCR to control the quality of the constructed library. Sequencing was performed using Illumina platform.

To quantify miRNA, we summarize the miRNAs identified in each sample, and calculate the TPM (tags per million) expression of each miRNA. The formula is as follows: $TPM=\frac{\text{T*}10^{6}}{\text{N}}$ [T stands for miRNA tags, N stands for total miRNA tags (Existing + existing edit + known + new predicted miRNA counts)], all miRNA expression profiles of all samples are obtained. In order to eliminate data noise, we filter out miRNAs with TPM< 1. The edgeR software was used for differential analysis of miRNA. The screening criteria for differential miRNAs is that *p* ＜ 0.05 and a fold change ≥2. We download all miRNA sequences and family information from TargetScan website (http://www.targetscan.org/).

### 2.3 Quantitative Real-Time PCR (qRT-PCR)

For mRNA and lncRNA detection, reverse transcription was performed with the Reverse Transcription Kit (Invitrogen, United States) according to the manufacturer’s protocol. Real-time quantitative PCR analysis was performed using the talent real-time PCR kit (TIAGEN, China). GAPDH was used as an endogenous control for lncRNAs and mRNA. PCR cycling conditions were conducted as follows: 95°C for 3 min; 40 cycles of 95°C for 5 s, and 60°C for 15 s. To analyze miRNA expression, cDNA synthesis was performed *via* the miRNA First-Strand cDNA Synthesis kit (TIANGEN, China). The reaction conditions were as follows: 42°C for 60 min, 95°C for 5 min. miRNA stem-loop primers were designed by BGI (Shenzhen, China), and the Real-Time primers were using Primer Premier 5.0 software based on the mature miRNA sequences. U6 small nuclear RNA served as endogenous controls. The quantification of miRNA was performed using the TaqMan™ miRNA Assay (cat. no. 4427975; Thermo Fisher Scientific, Inc.) RT-qPCR and thermocycling conditions were conducted as follows: 95°C 2 min; 40 cycles 95°C 15 s, 60°C 15 s and 68°C 30 s. The PCR amplification was performed in a GeneAmp PCR system 9700 Thermocycler (Applied Biosystems). SIX3OS1-shRNA and NC-shRNA lentiviruses were purchased from Guangzhou Aiji Biotechnology Co., Ltd. The primer sequences are detailed in Supplementary Table S1.

### 2.4 Function Enrichment Analysis

To assess functional enrichment, Gene Ontology (GO) Biological Processes term and Kyoto Encyclopedia of Genes and Genomes (KEGG) pathway analyses of mRNAs in the ceRNA network [The ceRNA hypothesis suggests that some RNAs, as ceRNAs, can regulate downstream mRNA’s expression by combining shared miRNAs. This hypothesis describes that ceRNAs can switch the function of target miRNAs by competing for the mRNA co-binding sites on the target miRNAs (Karreth and Pandolfi, 2013)] were performed using Cytoscape. GO enrichment analysis provides all GO terms that significantly enriched in ceRNAs comparing to the genome background, and filter the ceRNAs that correspond to biological functions. Firstly, all ceRNAs were mapped to GO terms in the Gene Ontology database (http://www.geneontology.org/), gene numbers were calculated for every term, significantly enriched GO terms in ceRNAs comparing to the genome background were defined by hypergeometric test. Genes usually interact with each other to play roles in certain biological functions. Pathway-based analysis helps to further understand genes biological functions. KEGG is the major public pathway-related database (http://www.kegg.jp/kegg/). Pathway enrichment analysis identified significantly enriched metabolic pathways or signal transduction pathways in ceRNAs compared with the whole genome background.

### 2.5 lncRNA-miRNA-mRNA Interaction Network Analysis

The potential functions of the expressed genes could be inferred through interaction networks (Salmena et al., 2011). To determine the correlation between miRNA-mRNA or miRNA-lncRNA. Firstly, the targeting relationship between miRNAs and candidate ceRNAs (lncRNA, mRNA) and the negative correlation relationship between expression levels were analyzed; then the positive correlation relationship between the expression levels of candidate ceRNAs was analyzed. Finally, candidate ceRNAs and their shared miRNA pairs for constructing ceRNA regulatory networks are obtained, that is, the lncRNA-miRNA-mRNA relationship pair. The networks were visualized using Cytoscape software (v3.6.0) (http://www.cytoscape.org/).

### 2.6 Neuron Culture

The culture of neurons was as previously described (Wang et al., 2018). In short, the hippocampus was isolated from P0 mice, stored in ice-cold Hank’s balanced salt solution, and incubated with 20 units/ml papain at 37°C for 30 min. The dissociated cells were suspended in the plating medium [DMEM/F12 (gibco) + 10% FBS (gibco)] and spread on the coverslips with PDL in a 24-well plate at a density of 30,000–60,000/cm^2^. After the initial incubation for 4 h, changed the medium to a maintenance medium [Neurobasal-Aedium (1x) (gibco) + B27 (gibco) + 1% GlutaMax (gibco) + 1% penicillin-streptomycin (Meilunbio)]. Neurons were kept in incubators with 37°C, 5% CO2. Replace half of the medium every 3 days.

### 2.7 Immunofluorescence

Neurons were washed with pre-cooled phosphate-buffered saline (PBS) once, following fixed with 4% paraformaldehyde (Meilunbio) for 1 h at room temperature. 0.1% Triton X-100 permeabilized neurons for three times (7 min each); then incubated with 3% Bovine Serum Albumin (Genview; FA016) for 1 h at room temperature; washed with PBS for three times (5 min each); and then incubated with anti-GFP (1:10,000; Abcam Ab290) in a humidified chamber overnight at 4°C; Neurons were incubated with Alexa FluorTM 488 secondary antibody (1:500, Thermo Fisher Scientific) was incubated for 1 h at room temperature the next day; after immersion for 3 times, it was mounted with DAPI-containing mounting medium. Observe under a laser confocal microscope, and use ImageJ software to determine the parameters.

### 2.8 Electrophysiology

The resistance of the glass electrode is 3～5 MΩ. When the electrode tip forms a GΩ seal with the neuron, the negative pressure sucks and breaks the cell, and then performs voltage clamp recording in the whole cell mode. The collected current was amplified by the amplifier multiclamp 700B, the software was Clampex10.5, and the data obtained were sorted and analyzed by Clampfit and miniAnalysis. The extracellular fluid contains NaCl 128 mmol/L, KCl 5 mmol/L, glucose 30 mmol/L, CaCl2 2 mmol/L, Hepes 25 mmol/L, MgCl2 1 mmol/L, and the pH is 7.3. Record microinhibitory synapses for post-current (mIPSC), add tetrodotoxin (TTX, 1 μM), 2-amino-5-phosphonovaleric acid (APV, 25 μM), and 6-cyano-7-nitroquine to the above extracellular fluid. Oxaline-2,3-dione (CNQX, 20 μM), intracellular fluid containing CsCl 140 mmol/L, EGTA 10 mmol/L, Hepes 5 mmol/L, CaCl2 2 mmol/L, MgATP 2 mmol/L, NaGTP 0.3 mmol/L, QX-314 5 mmol/L. When recording the tiny excitatory postsynaptic current (mEPSC), add TTX (1 μM) and PTX (100 μM) to the extracellular fluid; the intracellular fluid contains K-gluconate 125 mmol/L, ethylene glycol ditetraacetic acid (EGTA) 5 mmol/L, KCl 10 mmol/L, Tris-phosphocreatine 10 mmol/L, Hepes 10 mmol/L, NaGTP 0.5 mmol/L, MgATP 4 mmol/L, pH 7.3.

### 2.9 Statistical Analysis

GraphPad Prism 8.0.1 (Graphpad Software Inc.) was used for data analysis and graphing. Data were presented as mean ± s.e.m. A two-tailed Student’s t-test was used to evaluate the differences between groups. *p* < 0.05 was considered statistically significant.

## 3 Results

### 3.1 HE Mouse Model’s Identification

**FIGURE 1:**
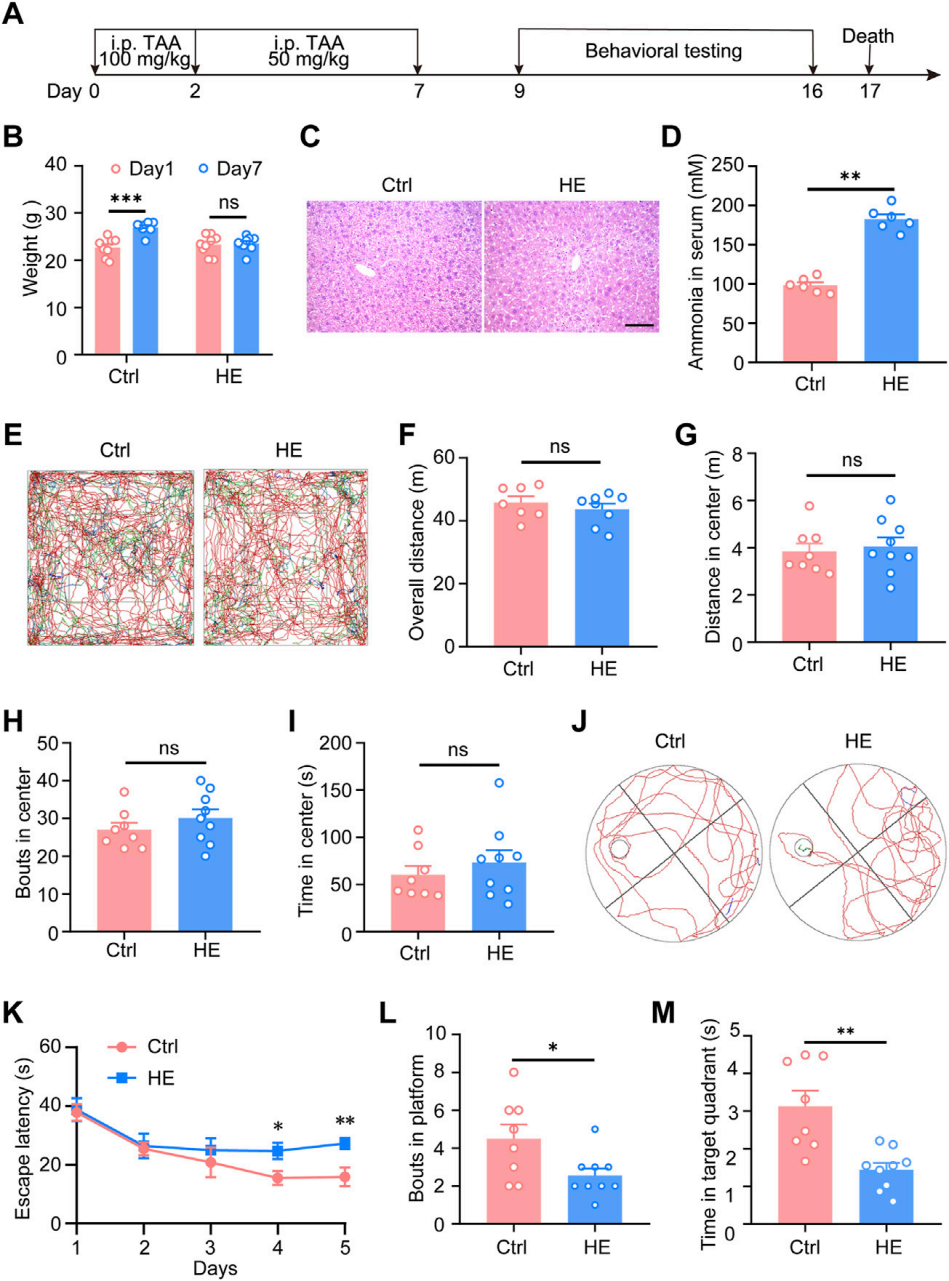
Modeling and identification of mice with hepatic encephalopathy. **(A)** Schematic of experimental procedures. At the age of 7 weeks, the HE mice received 100 mg/kg TAA intraperitoneal injections daily on the first and second days and then 50 mg/kg TAA daily from the third to the seventh day. From day 9 to day 16, perform behavioral tests, subsequently, the mice were sacrificed on day 17. **(B)** Body weight analysis of mice during the modeling period. **(C)** Representative photomicrographs of Hematoxylin-eosin staining in liver sections. Scale bar, 100 μm. **(D)** Changes in serum ammonia concentration in two groups of mice. **(E)** Representative traces of 10 min in open field tests. Effect of HE on total distance traveled **(F)**, central area movement distance **(G)**, bouts in the center **(H)**, and duration in the center **(I)** in the open-field test on mice (*n* = 8 mice for control; *n* = 9 mice for HE; *p* > 0.05). **(J)** Representative traces of Morris water maze test. **(K)** During the learning phase of the MWM, the escape latency to the platform of the two groups of mice was significantly different (*p* = 0.0035), where HE mice took significantly longer to find the platform than control mice. **(L)** Platform crossing times (*p* = 0.0307) and **(M)** time spent at target quadrant (*p* = 0.0014) were significantly lower than in the control group. Data were shown as mean ± SEM. *p < 0.05, **p < 0.01, ***p < 0.001; ns, no significant difference.

Before RNA-seq, HE mouse models were constructed and identified (Figure 1A). During the modeling period, the weight of HE mice increased slowly compared with control mice (Figure 1B). The lack of significant increase in body weight in HE mice indicated that TAA induced impaired liver function in mice, leading to decreased appetite, reduced diet, and malnutrition in HE mice, resulting in no significant increase in body weight. Compared to the control group, liver tissue in the HE group showed characteristic focal necrosis and inflammatory infiltration (Figure 1C). The serum ammonia levels of the two groups of mice were also detected. The results showed that the serum ammonia level of the HE mice increased significantly (Figure 1D). To evaluate the behavioral changes of HE mice, open field test was first performed (Figure 1E). The results showed that compared with control mice, there was no statistical difference in the total distance of movement, the distance moved in the central area, the number of passes through the center area, and the time spent in the center of HE group (Figures 1F–I). It suggested that HE mice had no motor dysfunction and no anxiety-like alteration. In addition, the Morris water maze test was used to examine the spatial learning and memory of mice (Figure 1J). Our results showed that compared with control group, HE mice took longer time to find the platform from the fourth day of training (*p* < 0.01; Figure 1K), and after the platform removal, platform crossing times and time spent at the target quadrant of HE group was significantly less than those of the control group (*p* < 0.05; Figures 1L,M). In the pre-experiment, we also conducted the elevated plus maze test, the Y-maze test, the novel-object recognition and the three-Chambered Social Test. The results showed that there were no differences in short-term memory, social and motor functions, and no anxiety-like alteration between the two groups of mice (Supplementary Figure S1). These results indicated that HE mice’s spatial learning and memory abilities were impaired, which corresponded to the clinical manifestations of hepatic encephalopathy patients. The evidence above data suggests that we have successfully established mouse models of hepatic encephalopathy. Subsequently, the hippocampal tissues of HE mice and control mice were used for RNA-seq.

### 3.2 Identification of lncRNA in Brain Tissue of Mice With Hepatic Encephalopathy

**FIGURE 2:**
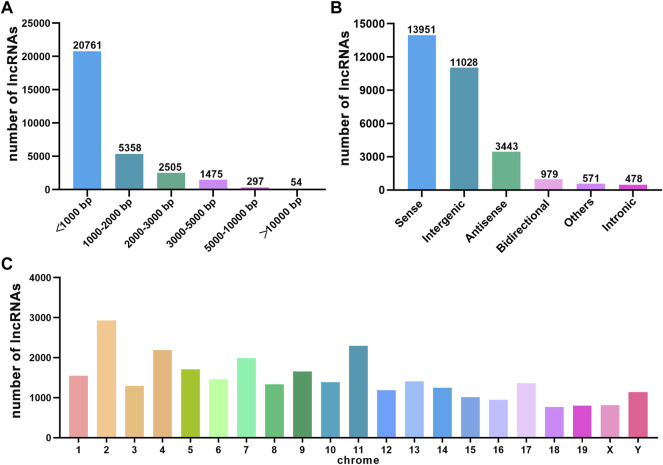
The characteristics of HE mouse lncRNAs detected by RNA sequence. **(A)** Length distribution of lncRNAs. **(B)** Classification of lncRNAs. **(C)** Chromosome distribution of lncRNAs.

We performed a whole transcriptomic analysis of the HE and the control groups to evaluate RNA expression differences (The data used herein has been deposited in NCBI’s Gene Expression Omnibus and are accessible through https://www.ncbi.nlm.nih.gov/bioproject/PRJNA804405). RNA sequencing of six cDNA libraries yielded more than 60 million original reads, most of which were clean reads, and more than 99.76% of clean reads were completely mapped to the reference mouse genome (Table 1). RNA-seq identified 30,450 lncRNAs. The average length of lncRNAs was 1121 bp, of which 86% were shorter than 2000 bp (Figure 2A). The classification of lncRNA included 11,028 (36%) intergenic, 13,951 (46%) sense, 979 (3%) bidirectional, 3,443 (11%) antisense, and 478 (2%) intronic and 571 (2%) other IncRNAs (Figure 2B). All detected lncRNAS were distributed among mouse chromosomes 1–19, X and Y, and lncRNAS had the largest number on the second chromosome (2,925, 10%) (Figure 2C).

**TABLE 1:** Summary of draft reads of six libraries by RNA-sequencing.

Sample	Raw reads	Clean reads (%)	Adapter (%)	Low quality (%)
Ctrl1	73256114	73076606 (99.75%)	23276 (0.03%)	156232 (0.21%)
Ctrl2	75434150	75233742 (99.73%)	27336 (0.04%)	173072 (0.23%)
Ctrl3	63054512	62893292 (99.74%)	25026 (0.04%)	136194 (0.22%)
HE1	78849186	78637562 (99.73%)	26680 (0.03%)	136194 (0.22%)
HE2	72976210	72780434 (99.73%)	25886 (0.04%)	169890 (0.23%)
HE3	81894380	81678406 (99.74%)	32746 (0.04%)	183228 (0.22%)

### 3.3 Differentially Expressed miRNAs, lncRNAs and mRNAs in HE Mice

**FIGURE 3:**
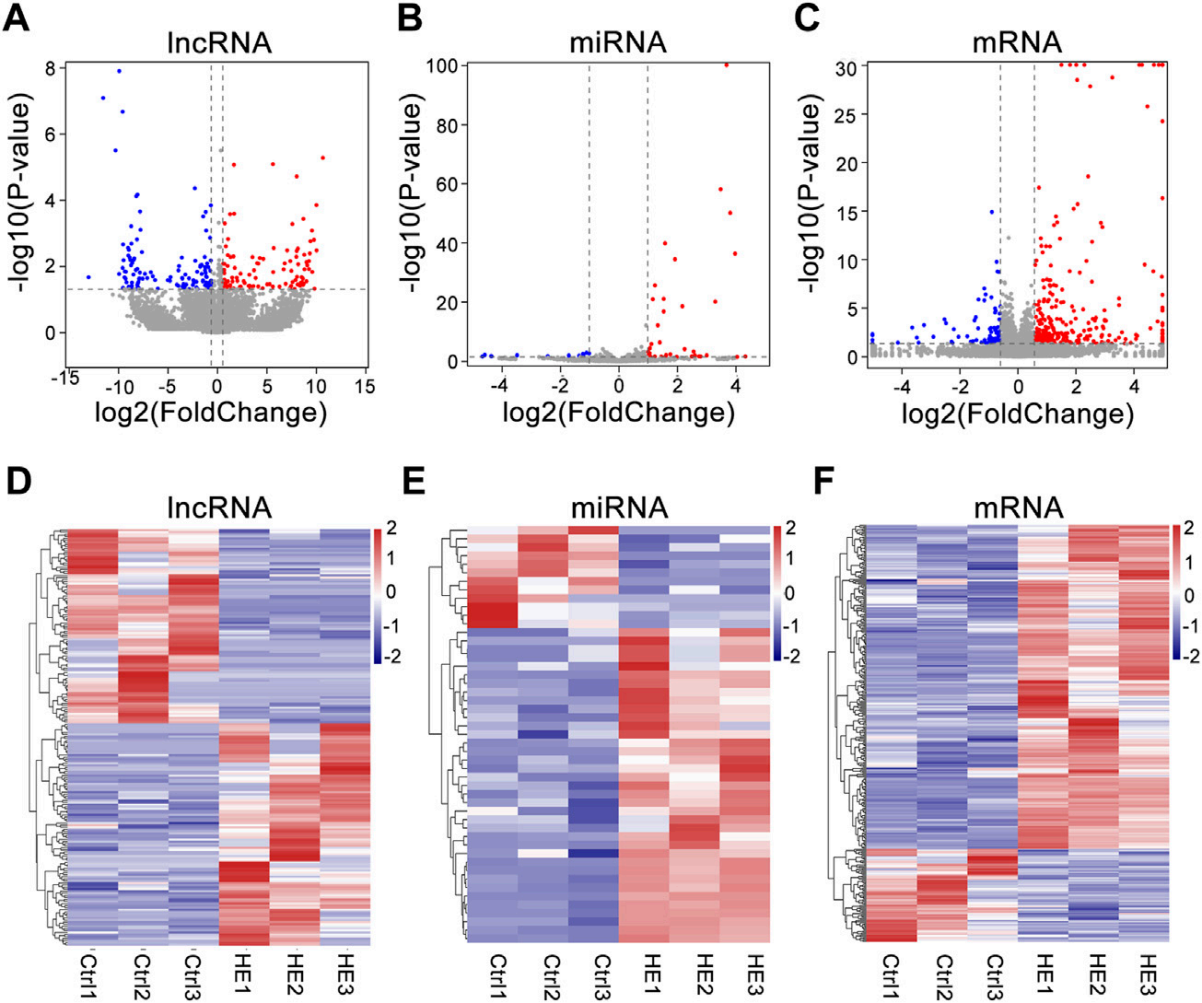
Expression Profiles of lncRNAs, miRNAs, and mRNAs in HE hippocampus tissues compared to normal tissues. **(A)** Volcano plot of differentially expressed lncRNAs. **(B)** Volcano plot of differentially expressed miRNAs. **(C)** Volcano plot of differentially expressed mRNAs. The vertical and horizontal coordinates respectively represent −log10 scaled (*p*-value) and log2-scaled fold changes. Horizontal dashed line indicates *p*-value < 0.05 and vertical dashed lines represent ≥2.0 × fold change. Red and blue dots represent upregulation and downregulation RNAs, respectively. **(D)** Heat map of differentially expressed lncRNAs. **(E)** Heat map of differentially expressed miRNAs. **(F)** Heat map of differentially expressed mRNAs. (Red color indicates upregulation; blue indicates downregulation).

We detected 30,450 lncRNAs in the hippocampus of the mouse by RNA-seq. Estimate the expression levels of various RNAs between the two groups based on the FPKM value. Compared with the control group, there were 229 lncRNAs siginificantly altered in HE mice (113 upregulated and 116 downregulated) (Supplementary Table S2). 49 known miRNAs were significantly altered (37 upregulated and 12 downregulated) (Supplementary Table S3). In addition, 282 mRNAs were upregulated in the HE group, while 81 were downregulated (Supplementary Table S4). The differential expression of lncRNAs, miRNAs, and mRNAs between the two groups was visually displayed by volcano and heat maps (Figure 3). In HE mice, the number of upregulated miRNAs and mRNAs were greater than that downregulated miRNAs and mRNAs, while the number of upregulated and downregulated lncRNAs were similar.

### 3.4 Validation of Gene Expression Profiles Using qRT-PCR

**FIGURE 4:**
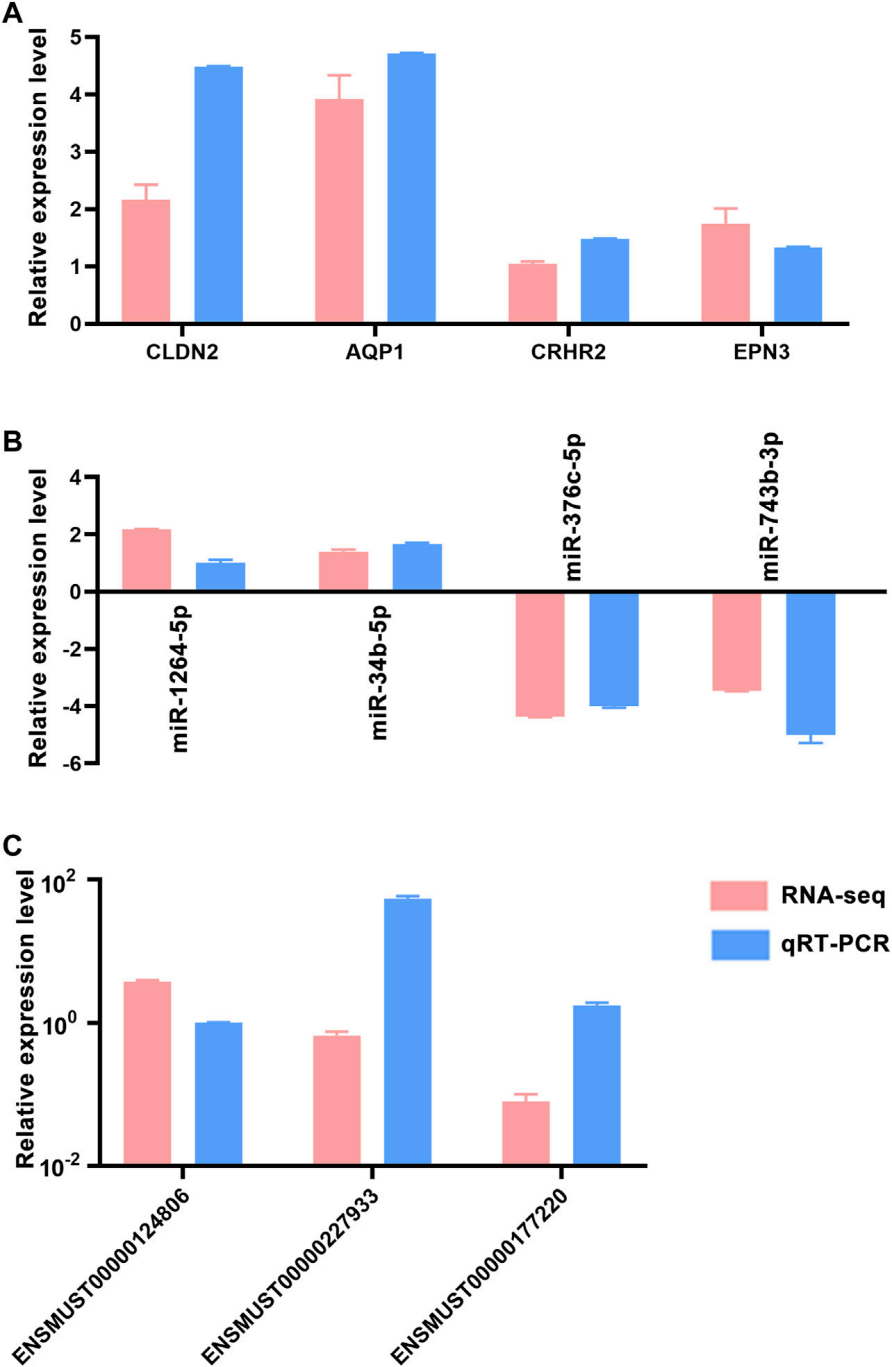
qRT-PCR validation of dysregulated RNAs. Comparison of log2 fold changes in mRNAs **(A)**, miRNAs **(B)** and lncRNAs **(C)** between RNA-Seq and qRT-PCR results. Error bars represented standard error of mean (SEM) (*n* = 5).

Quantitative real-time PCR (qRT-PCR) was used to confirm the accuracy and reproducibility obtained from RNA-seq analysis. We randomly selected four mRNAs (CLDN2, AQP1, CRHR2 and EPN3), four miRNAs (miR-1264-5p, miR-34b-5p, miR-376c-5p and miR-743b-3p) and three lncRNAs (ENSMUST00000124806, ENSMUST00000227933 and ENSMUST00000177220) for qRT-PCR analysis. The qRT-PCR results were consistent with the trend of RNA-seq data (Figure 4), which indicated that the RNA-seq results were reliable (Considering that we performed RNA-seq with three mice per group, this only met the minimum number of statistical analyses. Therefore, it is recommended that researchers verify the expression status of the genes to be studied before conducting experiments).

### 3.5 GO/KEGG Pathway Analysis of Differentially Expressed mRNAs

GO analysis was performed to elucidate the roles of differentially expressed mRNAs identified by RNA-seq. The results showed that differentially expressed mRNAs were significantly enriched in 51 GO entries. Upregulated mRNAs were primarily enriched in cellular process, single-organism process, signaling and behavior in biological processes; most of the cellular components were enriched in cell, synapse, organelle and membrane; molecular functions were mostly enriched in binding, catalytic activity and transporter activity (Supplementary Figure S2A). On the other hand, the downregulated mRNAs were primarily enriched in positive regulation of biological process, regulation of biological process; cell components were mostly enriched in cell, synapse part and macromolecular complex; molecular functions were mostly enriched in binding, nucleic acid binding transcription factor activity (Supplementary Figure S2B; Supplementary Table S5).

**FIGURE 5:**
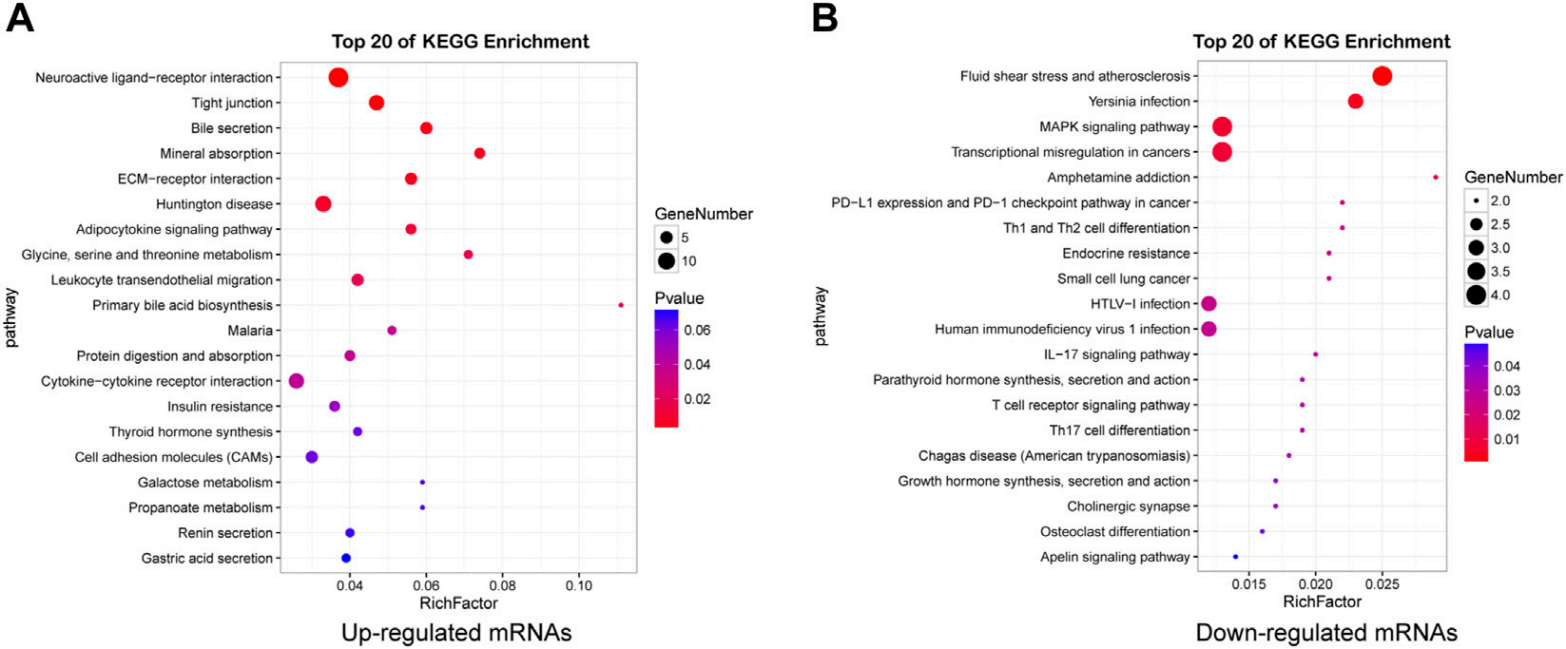
Detailed information for the top 20 KEGG pathways. Pathway enrichment analysis of upregulated **(A)** and downregulated **(B)** mRNAs in HE mice.

KEGG pathway analysis revealed that there were 117 pathways enriched in upregulated mRNAs and 23 enriched in downregulated mRNAs (Supplementary Table S6). Neuroactive ligand-receptor interaction, ECM-receptor interactions, Tight junction, Huntington disease, Glycine, serine and threonine metabolism, Cell adhesion molecules (CAMs), AMPK signaling pathway were the most enriched, indicating that dysregulated mRNAs may play important roles in nervous system dysfunction. The top 20 pathways of mRNAs are shown in Figures 5A,B (Supplementary Table S7).

### 3.6 Construction and Analysis of the Interaction Network

**FIGURE 6:**
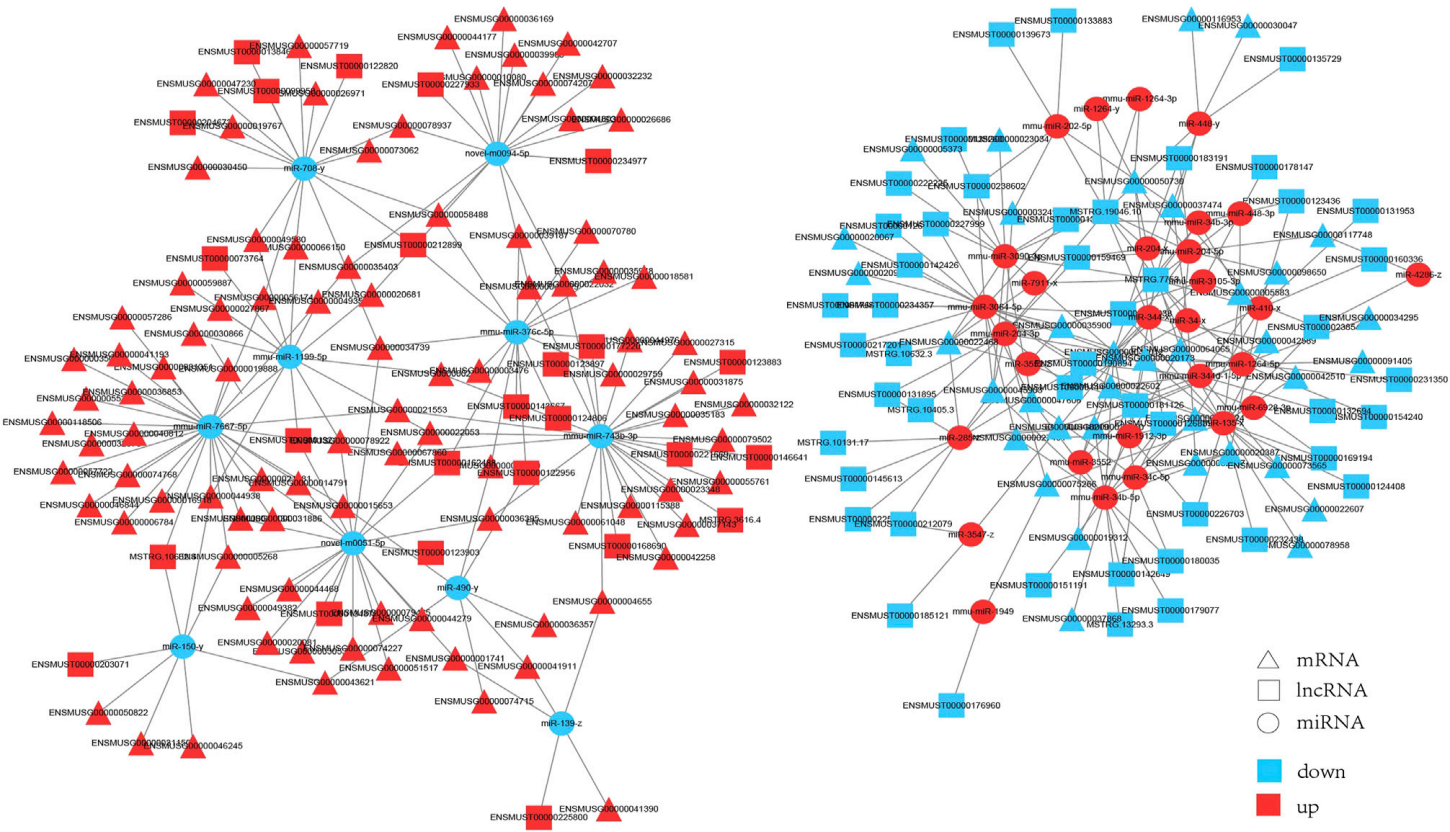
The interaction network of lncRNA-miRNA-mRNA. Triangle represents mRNAs, square represents lncRNAs, circle represents miRNAs. Red: upregulation; blue: downregulation.

We built interaction networks of lncRNA-miRNA-mRNA based on the expression profiles in HE mice. First, lncRNAs and mRNAs targeted by differentially expressed miRNAs were predicted, respectively. A total of 73 lncRNAs, 39 miRNAs and 134 mRNAs interactions were identified (Supplementary Table S8). We constructed co-expressed lncRNA-miRNA-mRNA visualization networks using cytoscape (v3.6.0) (Figure 6). In the networks,mmu-miR-7667-5p (degree = 33), miR-490-y (degree = 28) and mmu-miR-743b-3p (degree = 24) had more target mRNAs. In addition, mmu-miR-3064-5p (degrees = 14), miR-285-z (degrees = 12) and mmu-miR-34b-5p (degrees = 12) had more target lncRNAs. The results showed that a single mRNA or lncRNA could be correlated with one or more miRNAs and vice versa. Therefore, the interaction between lncRNAs, miRNAs and mRNAs may mediate the progression of HE.

**FIGURE 7:**
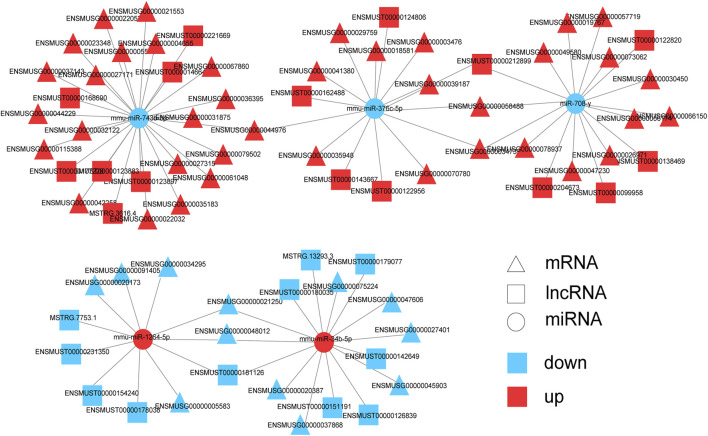
Predicted networks of mRNAs related to nervous system and correlative miRNAs and lncRNAs. Triangle represents mRNA, square represents lncRNA, and circle represents miRNA. Red: upregulated RNAs; blue: downregulated RNAs.

Next, mRNAs related to the nervous system and its associated lncRNAs, miRNAs were selected to produce ceRNA networks (Figure 7; Supplementary Table S9). The sub-network contained five core miRNA nodes (miR-743b-3p, miR-376c-5p, miR-708-y, mmu-miR-1264-5p, miR-34b-5p). It was predicted that Jade2, Fos and Npas4 were the target genes of miR-34b-5p. Aqp1 was the target gene of miR-743b-3p. According to KEGG analysis, these mRNAs were key genes for nervous system development, synaptic transmission, synaptic plasticity, and synapse assembly. The above results indicate that these lncRNAs might interact with miRNAs to regulate the expression of mRNAs’ expression and play important roles in nervous system development, synaptic transmission, and synaptic organization of HE mice.

### 3.7 Silencing of SIX3OS1 Improved Dendritic Spines Development and Synaptic Function

**FIGURE 8:**
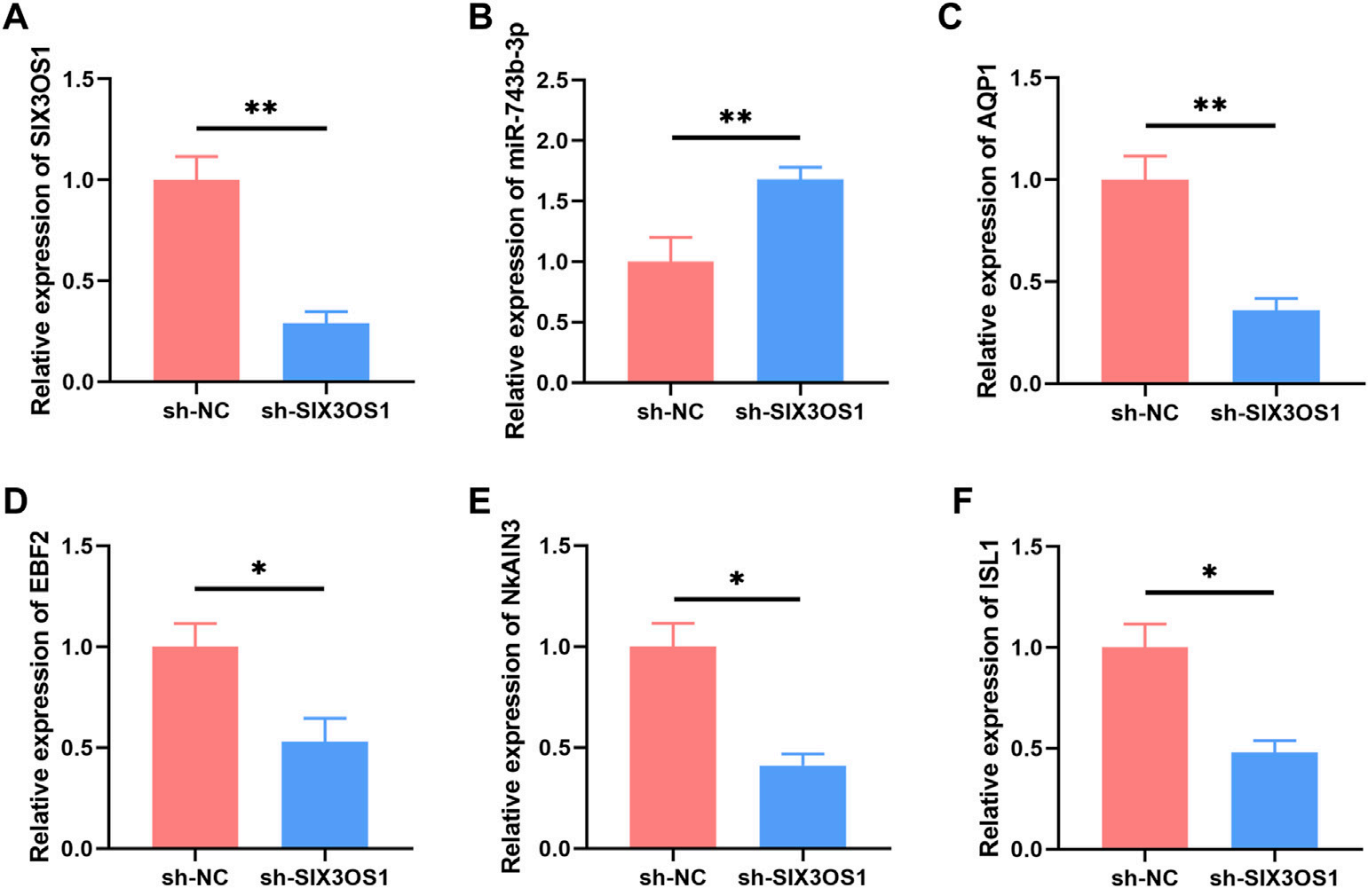
SIX3OS1 regulates the expression of miR-743b-3p, AQP1, EBF2, NKAIN3 and ISL1 in HE mice. **(A)** qRT-PCR confirmed that SIX3OS1-shRNA lentiviruses inhibited the expression of SIX3OS1. Silencing SIX3OS1 induces miR-743b-3p upregulate **(B)**, while AQP1 **(C)**, EBF2 **(D)**, NKAIN3 **(E)**, and ISL1 **(F)** downregulate. GAPDH was used as an internal control. Error bars: standard deviation (*n* = 3). qRT-PCR, quantitative reverse transcription-polymerase chain reaction. *p < 0.05, **p < 0.01.

**FIGURE 9:**
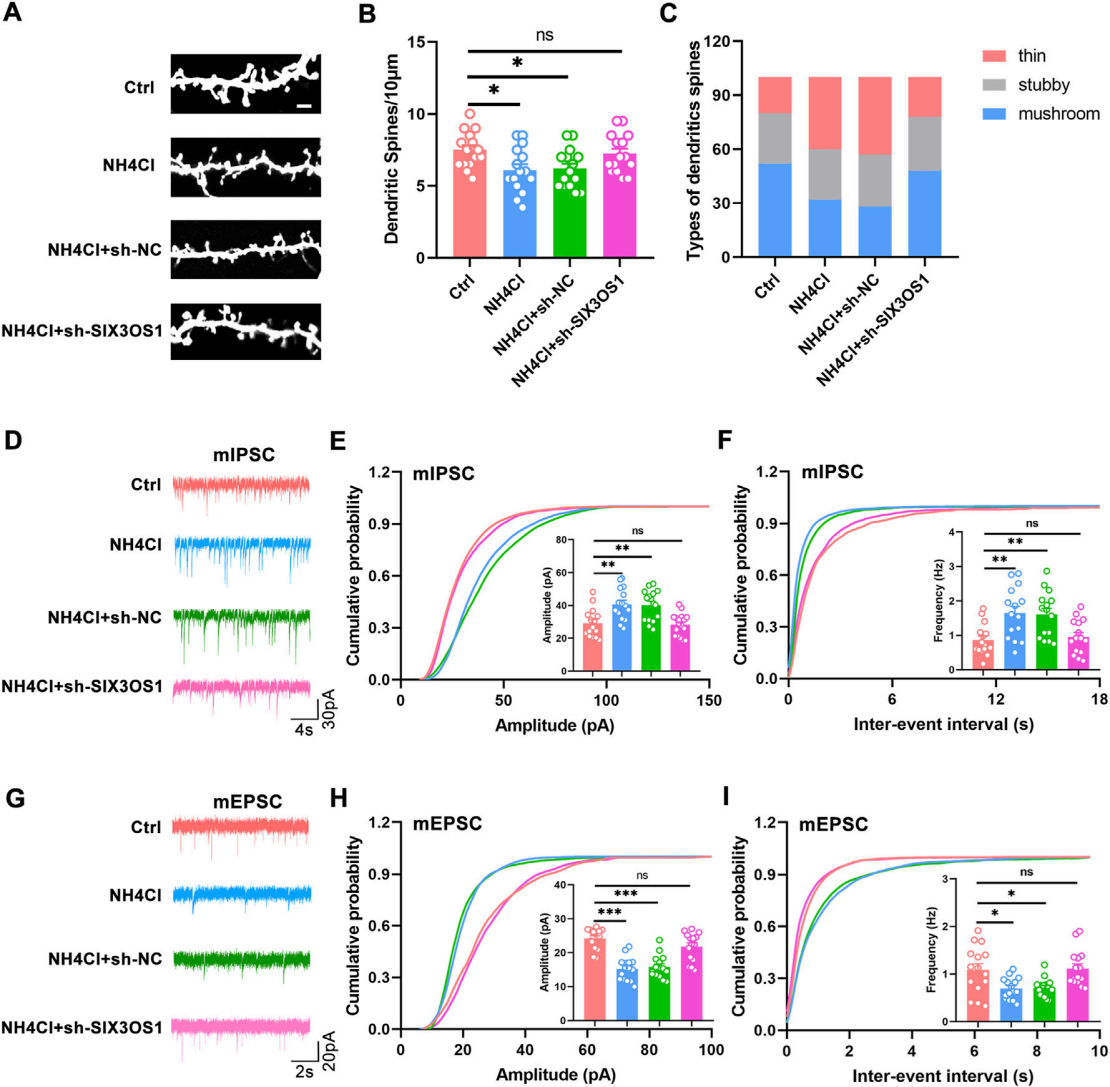
SIX3OS1 silencing improved dendritic spines’ maturity and synaptic function. **(A)** Representative immunofluorescence staining confocal images of dendrites of hippocampal neurons. Scale bar, 2 μm. **(B)** Dendritic spine density of NH_4_Cl-treated hippocampal neurons was reduced compared with control group. Dendritic spine density of NH_4_Cl + sh-SIX3OS1 group neurons was similar to that of control group. *n* = 15 neurons per group. one-way ANOVA followed by Tukey’s post-hoc test. **(C)** Mushroom-shaped dendritic spines of NH_4_Cl-treated hippocampal neurons was reduced compared with control group. Mushroom-shaped dendritic spines of NH_4_Cl + sh-SIX3OS1 group neurons was similar to that of control group. *n* = 15 neurons per group. **(D)** Representative mIPSC traces. **(E)** Increased mIPSC amplitude of hippocampal neurons in NH_4_Cl-treated. Reduced mIPSC amplitude of hippocampal neurons in NH_4_Cl-sh-SIX3OS1 treated. **(F)** Increased mIPSC frequency of hippocampal neurons in NH_4_Cl-treated. Reduced mIPSC frequency of hippocampal neurons in NH_4_Cl-sh-SIX3OS1 treated. **(G)** Representative mEPSC traces. **(H)** Reduced mEPSC amplitude of hippocampal neurons in NH_4_Cl-treated. Increased mEPSC amplitude of hippocampal neurons in NH_4_Cl-sh-SIX3OS1 treated. **(I)** Reduced mEPSC frequency of hippocampal neurons in NH_4_Cl-treated. Increased mEPSC frequency of hippocampal neurons in NH_4_Cl-sh-SIX3OS1 treated. *n* = 15 neurons per group. one-way ANOVA followed by Tukey’s post-hoc test. Data were shown as mean ± SEM. *p < 0.05, **p < 0.01, ***p < 0.001; ns, no significant difference.

In the ceRNA network, SIX3OS1 was one of the significantly upregulated lncRNAs. We constructed its shRNA lentiviral vectors and transduced them into cortex neurons *in vitro* to assess the roles of SIX3OS1 (The number of primary cultured hippocampal neurons is limited, and it is difficult to obtain enough samples for detection. Therefore, cortical neurons were used to verify the regulatory relationship between SIX3OS1 and its predicted targeted genes). SIX3OS1 silencing was confirmed by RT-PCR (Figure 8A). RT-PCR analysis of SIX3OS1-shRNA cells showed that the expression of miR-743b-3p targeted by SIX3OS1 was upregulated; AQP1, EBF2, NKAIN3 and ISL1 were downregulated (Figures 8B–F). We further explored the effects of SIX3OS1 on the dendritic spines and synaptic transmission of NH_4_Cl-treated neurons. The results showed that dendritic spine density and maturity of the NH_4_Cl and NH_4_Cl + sh-NC groups significantly decreased compared with the control group. The NH_4_Cl + sh-SIX3OS1 group was similar to the control group (Figures 9A–C).

Studies have shown that the imbalance of excitatory/inhibitory neurotransmitters is one of the major causes of HE (Rao, 2002; Nardone et al., 2016). Therefore, we measured the inhibitory and excitatory transmission of neurons transfected with sh-SIX3OS1 during hyperammonemia by whole-cell patch-clamp recordings. Compared with the control group, hyperammonemia significantly increased the amplitude and frequency of hippocampal neuron mIPSC, and decreased the amplitude and frequency of mEPSC. This change could be reversed after transfection with sh-SIX3OS1(Figures 9D–I). The above research showed that SIX3OS1 was essential for the maintenance of synaptic function.

## 4 Discussion

Many studies have shown lncRNA participates in liver diseases as well as plays crucial roles in various neurological diseases, such as liver, cancer (Wang et al., 2017), acute liver failure (Wang et al., 2020), Alzheimer’s disease (Zhou et al., 2019) as well as Parkinson’s disease (Cai et al., 2020). However, the precise contribution of lncRNAs to cognitive dysfunction in HE patients remains largely unknown. This study explored the lncRNA, miRNA and mRNA expression profiles of normal and HE hippocampus tissues to address this issue.

The literature has reported that the synaptic plasticity of HE mice has changed (Franca et al., 2019; Sun et al., 2019), which is consistent with the results of this study. GO analysis showed that many significantly different mRNAs were related to synaptic parts. In addition, the KEGG pathway analysis indicated that the significantly enriched pathways include neuroactive ligand-receptor interaction, Glycine, serine and threonine metabolism, AMPK signaling pathway. Llansola et al. (2015) believe that the cognitive function alteration in hepatic encephalopathy (HE) was the result of neurotransmission and neuronal network disorders. Blocking serotonergic signaling preferentially triggers synapses in the thalamic striatum in peak-time-dependent long-term depression (T-LTD) (Cavaccini et al., 2018). Our previous studies have shown that AMPA receptors play important roles in synaptic plasticity and synaptic function (Zhang et al., 2020; Cheng et al., 2022). Inhibition of AMPK/eEF2K/eEF2 signaling pathway improves synaptic function in SAMP8 mice (Dong et al., 2019). These studies demonstrate that the genes we screen for through the HE model are indeed closely related to nervous system function, especially synaptic function.

To date, increasing evidence supports that competitive endogenous RNA networks play important mechanisms in explaining the post-transcriptional regulation of genes (Tay et al., 2014). We built interaction networks of lncRNA-miRNA-mRNA based on the dysregulated RNAs in HE mice further to understand the role of lncRNAs via calculation and inference. The networks include 73 lncRNAs, 39 miRNAs, and 134 mRNAs. Among them, mRNAs AQP1, CLDN2, CRHR2, and EPN3 related to the nervous system were upregulated, while other related genes such as CUX2, ARC, and NPAS4 were downregulated. Studies have shown that neuron PAS domain protein 4 (NPAS4) was gene related to long-term synaptic plasticity and have significant regulatory effects on memory (Heroux et al., 2018). In addition, NPAS4 plays a role in the balance of excitatory and inhibition (Unno et al., 2020). In summary, the differentially expressed lncRNAs and its target gene networks may play an important role in maintaining the cognitive function of the nervous system. AQP1 is one of the most significantly expressed genes, so we further explored AQP1.

SIX3OS1 is one of the most important lncRNAs in our lncRNA-miRNA-mRNA interaction networks; it was predicted that SIX3OS1 is related to miR-743b-3p and AQP1. AQP1 is an aquaporin, and it’s upregulation led to increased water influx and disturbance of brain homeostasis (Trillo-Contreras et al., 2019). In addition, studies have shown that AQP1 is significantly upregulated in AD mice. It promotes neuronal apoptosis by inhibiting the Wnt signaling pathway, thereby impairing learning and memory. AQP1 silencing has a protective effect on hippocampal neurons of AD mice, thereby improving the cognitive function of AD mice (Yu et al., 2020). SIX3OS1 affects neurons and decreases glial cells’ differentiation (Ramos et al., 2013), as well as SIX3OS1 can activate the AKT signaling pathway by up-regulating Fezf1 (Zou et al., 2020). Therefore, we believe that SIX3OS1 and AQP1 play a vital role in HE-induced cognitive dysfunction in mice.

Furthermore, we verified the effects of SIX3OS1 on neuronal function by silencing SIX3OS1. Consistent with our prediction, SIX3OS1 silencing upregulated the expression level of miR-743b-3p and decreased the expression level of AQP1. SIX3OS1 silencing not only improved the abnormal development of dendritic spines caused by hyperammonemia, and corrected the synaptic neurotransmission disorder caused by hyperammonemia. LncRNA H19 acts as a ceRNA of mir-19a-3p to target PTEN and promote cerebral ischemia/reperfusion injury via PI3K/AKT pathway (Gao et al., 2020). LncRNA SNHG3 functions as ceRNA to sponge microRNA-215 to up-regulate ATG7 expression, promoting autophagy-induced neuronal cell apoptosis (Cao et al., 2020). Especially, research has shown that Rpph1 can increase the expression of CDC42 by competing with miR-330-5p, thereby promoting hippocampal neuron dendritic spine formation (Cai et al., 2017). In summary, we hypothesized that SIX3OS1 may regulate the expression of AQP1 through targeted binding of miR-743b-3p, thereby causing learning and memory dysfunction. However, the specific regulation mechanism needs to be further studied.

## 5 Conclusion

In conclusion, the present study revealed many dysregulated lncRNAs, miRNAs and mRNAs, which may be related to the development process of impaired learning and memory function in HE. SIX3OS1 may act as a ceRNA of miR-743b-3p to target AQP1 and regulate synaptic function, thus leading to HE-induced memory dysfunction. Further studies are needed to determine if modulating these lncRNAs can be therapeutically beneficial in HE.

## Data Availability

The datasets presented in this study can be found in online repositories. The names of the repository/repositories and accession number(s) can be found below: NCBI SRA BioProject, accession no.: PRJNA804405.

## Supplementary Material

Supplementary Figure S1 HE mice did not show difference in motor function, short-term memory, and social interaction. In the elevated plus maze test, the time spent with open arms **(A)**, the number of times to enter the open arms **(B)**, the time spent in closed arms **(C)**, and the total distance **(D)** were not statistically different between the two groups of mice. **(E)** In the Y-maze test, there was no statistical difference in the rate of free alternation between the two groups of mice. **(F)** In the novel object recognition test, the sniffing time of two identical objects was not statistically different between the two groups of mice during the training phase. **(G)** Both groups of mice sniffed the novel object significantly longer than the familiar object during the test phase. **(H)** In the three-chamber social interaction test, HE and control mice had significantly longer sniffing time to the stranger mouse (S1). **(I)** The social preference index of HE mice was not significantly different from control mice. **(J)** HE mice also showed significantly longer sniffing time to another stranger mouse (S2) compared with control mice. **(K)** The social novelty index of HE mice was not significantly different from control mice.

Supplementary Figure S2 GO analysis of differentially expressed mRNAs. GO analysis of upregulated **(A)** and downregulated **(B)** mRNAs in HE mice.
