# Supplementary material for: Comprehensive Analysis of lncRNAs, miRNAs and mRNAs in Mouse Hippocampus With Hepatic Encephalopathy
Source: Front Genet. 2022 May 5;13:868716. doi: 10.3389/fgene.2022.868716 (PMC9117740; doi:10.3389/fgene.2022.868716)
Supplement: Supplementary file 1 [file DataSheet1.ZIP › Table S1 Primers designed for qRT-PCR.docx]

# Supplementary Materials

## Supplementary Tables

Supplementary Table S1. Primers of mRNAs, miRNAs and lncRNAs for qRT-PCR.

| mRNA / lncRNA / miRNA | Primer sequences (5'-3') |
| --- | --- |
| Cldn2-F | ATGCCTTCTTGAGCCTGCTT |
| Cldn2-R | CAGTGTCTCTGGCAAGCTGA |
| Aqp1-F | TCCAGGACAACGTGAAGGTG |
| Aqp1-R | CCGGAGGATGCTGATCTGAC |
| Crhr2-F | ACCGAATCGCCCTCATTGTC |
| Crhr2-R | GTCGATGAGTTGCAGCAGGA |
| Epn3-F | CTTTCCTCCCGAGAGTGCTG |
| Epn3-R | TACCTTGGACCGAAAGGCAC |
| EBF2-F | CCTCGTGTCTCTAACGCAGG |
| EBF2-R | AAGCATCACCTGTTCTTTGCAG |
| NKAIN3-F | GAACTAGCCCCAAGCTGTGA |
| NKAIN3-R | GCTGATGGGTATCCACGGAG |
| ISL1-F | GTAGCATCGATGTCCTCGCA |
| ISL1-R | CCCACTTTCTCCAACAGGGG |
| m-ENSMUST00000124806-F | CCCAGACTGATCTTGCTTCCTT |
| m-ENSMUST00000124806-R | GCGTATTGGTGAAATGTCGG |
| m-ENSMUST00000227933-F | GGGGATGATGACCCTCCTGA |
| m-ENSMUST00000227933-R | AGGTAACACAGGTGACGCAG |
| m-ENSMUST00000177220-F | GGTGGGTCTAGGGTCACTGC |
| m-ENSMUST00000177220-R | CAGCCACGAGGGCTATTTGT |
| M-Gapdh_F | GTGTTCCTACCCCCAATGTGT |
| M-Gapdh_R | ATTGTCATACCAGGAAATGAGCTT |
| miR-1264-5p-R | GTCGTATCCAGTGCGTGTCGTGGAGTCGGCAATTGCACTGGATACGACAACAAAT |
| miR-1264-5p-F | aggTccTcaaTaagTaTTT |
| miR-34b-5p-R | GTCGTATCCAGTGCGTGTCGTGGAGTCGGCAATTGCACTGGATACGACACAATCA |
| miR-34b-5p-F | aggcagTgTaaTTagcTg |
| miR-376c-5p-R | GTCGTATCCAGTGCGTGTCGTGGAGTCGGCAATTGCACTGGATACGACTAAACAT |
| miR-376c-5p-F | Ggtggatattccttctatg |
| miR-743b-3p-R | GTCGTATCCAGTGCGTGTCGTGGAGTCGGCAATTGCACTGGATACGACTCTATTC |
| miR-743b-3p-F | GAAAGACATCATGCTGAAT |
| m/h-u6-F | CTCGCTTCGGCAGCACA |
| m/h-u6-R | AACGCTTCACGAATTTGCGT |
| UR | CAGTGCGTGTCGTGGAGT |
