# Supplementary material for: Comprehensive Analysis of lncRNAs, miRNAs and mRNAs in Mouse Hippocampus With Hepatic Encephalopathy
Source: Front Genet. 2022 May 5;13:868716. doi: 10.3389/fgene.2022.868716 (PMC9117740; doi:10.3389/fgene.2022.868716)
Supplement: Supplementary file 1 [file DataSheet1.ZIP › Supplementary Material.docx]

Supplementary Material

# Supplementary Tables

**Supplementary Table 1.** Primers used for qRT-PCR in this study.

**Supplementary Table 2.** Differentially expressed lncRNA transcripts from RNA-seq analysis. Information about the upregulated and downregulated lncRNA transcripts in HE mice compared with control mice.

**Supplementary Table 3.** Differentially expressed miRNA transcripts from RNA-seq analysis. Information about the upregulated and downregulated miRNA transcripts in HE mice compared with control mice.

**Supplementary Table 4.** Differentially expressed mRNA transcripts from RNA-seq analysis. Information about the upregulated and downregulated mRNA transcripts in HE mice compared with control mice.

**Supplementary Table 5.** GO analysis results.

**Supplementary Table 6.** KEGG analysis results.

**Supplementary Table 7**. The top 20 KEGG pathways analysis results

**Supplementary Table 8.** Information about lncRNA-miRNA-mRNA co-expression network.

**Supplementary Table 9.** Predicted ceRNA networks of mRNAs related to nervous system and correlative miRNAs and lncRNAs
